# Characterising the enzyme-driven metabolic shifts in rancid pearl millet flour using metabolomics approaches: a step towards improving quality and shelf-life

**DOI:** 10.3389/fnut.2025.1691522

**Published:** 2025-10-23

**Authors:** Ranjeet R. Kumar, Ashok Kumar, T. Vinutha, Suneha Goswami, Sudhir Kumar, Sumerpal Singh, C. T. Manjunath Prasad, Gyan P. Mishra, Jasdeep C. Padaria, Girish K. Jha, Tara Satyavathi Chellapilla, Viswanathan Chinnusamy

**Affiliations:** ^1^Division of Biochemistry, ICAR-Indian Agricultural Research Institute, New Delhi, India; ^2^Division of Plant Physiology, ICAR-Indian Agricultural Research Institute, New Delhi, India; ^3^Division of Genetics, ICAR-Indian Agricultural Research Institute, New Delhi, India; ^4^Division of Seed Sciences and Technology, ICAR-Indian Agricultural Research Institute, New Delhi, India; ^5^ICAR-National Institute of Plant Biotechnology, Pusa Campus, New Delhi, India; ^6^ICAR-Indian Agricultural Statistics Research Institute, Pusa Campus, New Delhi, India; ^7^ICAR-Indian Institute of Millet Research, Hyderabad, India

**Keywords:** metabolomics, rancidity, pearl millet, lipid oxidation, flour

## Abstract

Rancidity significantly limits the shelf-life and market potential of pearl millet flour and its processed food items, despite the grain’s nutritional superiority. This study employed a comprehensive metabolomic and biochemical approach to unravel the mechanisms underlying rancidity in pearl millet cultivars—Pusa-1201 (hybrid) and Chadhi Bajri (landrace). Untargeted LC–MS profiling revealed pronounced metabolic alterations in stored flour of Pusa-1201, with elevated levels of lipid degradation products (3-oxotetradecanoyl-CoA), pigments (chlorophyllide b), free fatty acids, etc. In contrast, Chadhi Bajri exhibited unique antioxidant and stress-protective metabolites (quercetin 3-sulfate, rosmarinate), indicating greater resistance to oxidative degradation. Enzymatic assays showed a storage-dependent increase in lipase, lipoxygenase, peroxidase, and polyphenol oxidase activities—particularly in Pusa-1201—correlating strongly with rancidity indices, including acid value (AV), peroxide value (PV), and free fatty acid content (FFA). Multivariate statistical analyses (PLS-DA, VIP plots, heat-maps) highlighted discriminatory metabolites contributing to rancidity progression and oxidative stress. A set of 25 metabolites, including phytol, ethanolamine, chlorophyllide b, and glucoside derivatives, emerged as key biomarkers of rancidity. These findings provide valuable insights for developing metabolite-based sensors to assess rancidity behaviour across diverse pearl millet accessions. Further, the information can be used to develop technology for enhancing flour’s stability and utilisation in developing processed food products.

## Introduction

Pearl millet (*Pennisetum glaucum*), is an exceptionally resilient and globally important cereal grain crop (1). It has very high tolerance to drought and heat stresses, and has inherent ability to grow in poor soils. It is considered as ‘nutricereal’ or ‘supergrain’, because of high grain nutrient density and wide ecological adaptability (2). It provides substantial energy and a significant protein content ranging from 9% to 13%, along with dietary fibre (8–11.5%) that aids in digestion and satiety. Pearl millet is rich in fats (5–5.4%), with 15–20% saturated fatty acids and 70–80% unsaturated fatty acids. Unsaturated fatty acids comprises of 25% mono-unsaturated fatty acids and 50–55% polyunsaturated fatty acids, including linoleic acid (3). It is an excellent source of micronutrients, such as iron, zinc, and calcium, and provides vitamin B and other antioxidants. These rich nutritional profiles contribute to a range of health benefits, including better glycemic control, enhanced cardiovascular health, and overall wellness (4).

Despite its nutritional supremacy and climate resilience, the popularisation and utilisation of pearl millet are significantly hindered due to the rapid degradation of its milled flour. This hydrolytic and oxidative deterioration leads to an undesirable development of off-flavours and odours, severely reducing the flour’s shelf-life (5). The primary culprits behind this degradation are two interconnected biochemical processes—enzymatic rancidity and oxidative rancidity. Understanding the mechanisms underlying off-odour development and oxidation of lipids will help in developing effective mitigation strategies (6).

Enzymatic rancidity is predominantly triggered by the activity of lipase, which hydrolyses triglycerides into free fatty acids, directly contributing to hydrolytic rancidity (7). Pearl millet is known to possess higher lipid content (~5–6%) and high lipase activity compared to many other cereal grains, accelerating the process of rancidity. On the other hand, lipoxygenase (LOX) catalyses the oxidation of polyunsaturated fatty acids into unstable hydroperoxides, which subsequently gets converted into highly volatile compounds like aldehydes and ketones (8). These breakdown oxidised products are largely responsible for the off-odour and bitter tastes associated with rancid flour. The high content of polyunsaturated fatty acids (PUFAs) in pearl millet, along with high activity of indigenous enzymes, renders it particularly susceptible to rapid spoilage. Therefore, mitigating these enzymatic and oxidative processes is important in extending the shelf-life of pearl millet flour. Development of technology to enhance the shelf-life will help in realising the full potential of this grain crop as a widely consumed, nutritious, and sustainable food source (9).

Metabolome profiling is an advanced omics technology involved in identification and analysis of the entire set of small-molecule metabolites (the metabolome) present within a biological system, such as grains (10). Metabolites represent the final products of enzymatic reaction and are directly influenced by both genetic makeup and environmental factors. It shows physiological state and phenotypic characteristics of a grain (11). This powerful approach utilises sophisticated analytical platforms like mass spectrometry (MS) coupled with gas or liquid chromatography (GC–MS, LC–MS) and nuclear magnetic resonance (NMR) spectroscopy. The advance metabolome technology are used to identify and quantify a vast array of compounds like amino acids, sugars, lipids, organic acids, vitamins, and specialised secondary metabolites (12). Metabolome profiling is becoming an indispensable tool in grain research, enabling deeper insights into grain quality attributes, nutritional composition, responses to biotic and abiotic stresses (drought, heat, pests), and the complex interplay between genotype, environment, and ultimate grain phenotype (13).

The study on rancidity in pearl millet has several practical consequences that can directly benefit both producers and consumers. By identifying the role of lipase activity in lipid degradation, strategies can be developed to control enzymatic rancidity, thereby extending the shelf-life of pearl millet flour without compromising its quality (7). Preservation of essential fatty acids through reduced rancidity will ensure better nutritional value of the flour, offering healthier products to consumers. The findings will also provide valuable guidance for selecting low-rancidity cultivars, enabling farmers and breeders to optimise both storage stability and marketability (2). Ultimately, reducing rancidity will enhance consumer safety and acceptability and lays the foundation for developing rapid metabolite-based sensors to monitor flour quality, supporting both industry standards and household use (14).

The present investigation of systematic identification of important metabolic signatures will provide deeper understanding of the specific compounds contributing to off-odours, and bitterness in flour paving the way for the development of accurate diagnostic markers for rancidity in flour and food items and will in turn facilitate the breeding of pearl millet lines with improved shelf-life.

## Materials and methods

Freshly harvested grains of pearl millet representing two distinct genetic backgrounds—a landrace (Chadhi Bajri) and a high-yielding hybrid (Pusa 1201)—were procured from the Division of Genetics, ICAR-Indian Agricultural Research Institute (IARI), New Delhi, India. The freshly harvested grains were manually cleaned to remove debris and non-viable seeds, and subsequently milled using a standardised pre-fabricated grain mill under controlled conditions to ensure uniform flour particle size. To simulate natural post-milling storage conditions and induce spontaneous rancidity, the freshly milled flour was transferred into translucent polypropylene bottles equipped with perforated caps. These perforations permitted controlled aeration, facilitating simultaneous oxidative and hydrolytic deterioration. The samples were then stored under ambient laboratory light conditions at a constant temperature of 35 ± 1 °C to mimic accelerated shelf-life degradation. Elevated temperature and light acts as stressors that promote lipid oxidation, enzymatic activity, and pigment degradation, thereby allowing rapid detection of rancidity-related biochemical and metabolic changes within a shorter experimental time frame (15). This design enabled the activation of native enzymes, including lipase, lipoxygenase (LOX), peroxidase (POX) and polyphenol oxidase (PPO), and promoted the oxidation of polyunsaturated fatty acids—closely simulating flour storage conditions, though need to be validated under realistic household or warehouse storage to ensure translational relevance. The flours were sampled at two key time points: M_0_—immediately after milling (fresh flour used as control), M_1_—after 30 days of storage (designated as rancid flour), and M_2_—after 60 days of storage (designated as severe rancid flour). These time points were selected based on preliminary kinetic data indicating a clear onset of rancidity symptoms (off-odour, elevated peroxide and acid value within 10 days under the applied conditions (14). Both fresh (0 DAM) and rancid flour (30 days after milling) samples were subsequently subjected to metabolomic profiling to investigate rancidity-associated metabolic changes. The samples collected at M_0_, M_1_, and M_2_ were used for the characterisation of biochemical traits linked with rancidity.

## Liquid chromatography–mass spectrometry (LC–MS) based metabolome profiling of fresh and rancid flour

### Sample preparation and metabolite extraction

Pearl millet flour samples (freshly milled and naturally aged samples) were accurately weighed (50 mg) into pre-chilled 2 mL micro-centrifuge tubes. A 10 μL aliquot of a mixed internal standard solution [containing 2-chlorophenylalanine (100 μM); D4-glutamic acid (50 μM)] was spiked into each sample. Metabolites were extracted by adding 400 μL of ice-cold 80% methanol in water. The samples were vortexed vigorously for 30 s followed by agitation using a bead mill homogeniser (Bead Ruptor 24) at 30 Hz for two cycles of 30 s, with 5 s rest between cycles, while keeping the samples on ice followed by centrifugation at 14,000 × *g* for 10 min at 4 °C. The supernatant (polar extract) was carefully transferred and to the remaining pellet, 400 μL of ice-cold chloroform was added, followed by vortexing, agitation and centrifugation. The dried extracts were reconstituted in 200 μL of acetonitrile:isopropanol:water (65:30:5, v/v/v) containing 0.1% formic acid and was transferred to an LC–MS vial for analysis.

### LC–MS metabolome profiling

Metabolome profiling was performed using a liquid chromatography-mass spectrometry (LC–MS) system (Vanquish Flex UHPLC system coupled to Orbitrap, Thermo Fisher Scientific). Acquity UPLC BEH C18 column (2.1 × 100 mm, 1.7 μm; Waters Corporation) was used for the chromatographic separation. The mobile phase consist of solvent A (0.1% formic acid in water) and solvent B (0.1% formic acid in acetonitrile). We have followed the standard gradient elution and the flow rate was maintained at 0.3 mL/min. Detection was conducted using electrospray ionisation (ESI) in both positive and negative modes, with data acquisition in full scan MS mode over an m/z range of 50–1,000.

### Data processing and statistical analysis

Raw LC–MS data were processed using Compound Discoverer 3.3 (Thermo Fisher Scientific). Metabolites were identified by comparing high-resolution mass data, retention times, isotope distributions, and MS/MS fragmentation patterns against both public databases (Metlin) and proprietary spectral libraries. MetaboAnalyst 5.0 was employed for statistical analysis. One-way ANOVA followed by Tukey’s HSD post-hoc test was applied to determine significantly altered metabolites among different sample groups. Orthogonal Partial Least Squares Discriminant Analysis (OPLS-DA) was performed for multivariate analysis to highlight key differentiating metabolites. Metabolites were considered significant if they had a VIP (variable importance in projection) score greater than 1.0 and an FDR-adjusted *p*-value below 0.05. Furthermore, pathway enrichment analysis was carried out in MetaboAnalyst 5.0 to identify metabolic pathways associated with the off-odour development and rancidity.

### Establishing the rancid behaviour of flour using biochemical traits

#### Determination of acid value (AV)

The acid value (AV) of pearl millet flour samples was determined based on a modified AOCS Official Method (16) quantifying the free fatty acids (FFAs) indicative of hydrolytic rancidity. Initially, oil was extracted from accurately weighed 5 g aliquots of homogenised flour using cold hexane extraction, where samples were vigorously shaken with 50 mL of hexane for 1 h, followed by centrifugation at 4000 × *g* for 10 min. The supernatant was carefully decanted, and the hexane was evaporated under a gentle stream of nitrogen gas to yield the crude oil, which was then stored at −20 °C until analysis. For AV determination, 2.5 g of the extracted oil was accurately weighed into a 250 mL Erlenmeyer flask, to which 125 mL of freshly neutralised solvent mixture (1:1 v/v isopropyl alcohol: toluene, neutralised with 0.1 M KOH using phenolphthalein indicator) was added to dissolve the oil. After adding 0.5 mL of 1% phenolphthalein indicator, the solution was immediately titrated with standardised 0.1 M ethanol KOH solution until a faint, permanent pink colour persisted for at least 30 s. A blank titration was performed concurrently using only the neutralised solvent mixture. The acid value was calculated using the formula: AV=W (*V*_s_−*V*_b_) × M × 56.1, where *V*_s_ and *V*_b_ are the volumes of KOH solution used for the sample and blank, respectively (mL), M is the molarity of KOH solution (mol/L), 56.1 is the molecular weight of KOH (mg/mmol), and W is the weight of the oil sample (g). All measurements were conducted in triplicate.

#### Determination of peroxide value (PV)

The peroxide value (PV) of the extracted pearl millet oil samples, indicative of primary oxidative rancidity products, was determined using a modified iodometric titration method based on AOCS Official Method (17). Oil samples, obtained via hexane extraction, were accurately weighed (5.00 ± 0.05 g) into a 250 mL glass-stoppered Erlenmeyer flask. The oil was dissolved by adding 30 mL of a freshly prepared 3:2 (v/v) acetic acid-isooctane solution. Immediately thereafter, 0.5 mL of fresh saturated potassium iodide solution was added. The flask was quickly stoppered, gently swirled, and allowed to stand in the dark for precisely 1 min with intermittent vigorous shaking. Following the reaction period, 30 mL of distilled water was added, and the liberated iodine was immediately titrated with a standardised 0.1 N sodium thiosulfate solution. As the yellow iodine colour faded, approximately 0.5 mL of 1% starch indicator solution was added, causing the solution to turn blue. Titration continued dropwise with vigorous agitation until the blue colour completely disappeared and remained colourless for at least 30 s. A blank titration was performed by following the same procedure without the oil sample. The peroxide value was calculated using the formula: PV=W (*V*_s_−*V*_b_) × M × 1,000, where *V*_s_ and *V*_b_ are the volumes of sodium thiosulfate solution used for the sample and blank (mL), M is the molarity (normality) of sodium thiosulfate solution (mol/L or eq/L), 1,000 is a conversion factor to kg, and W is the weight of the oil sample (g). All determinations were performed in triplicate.

### Estimation of free fatty acids (FFAs)

Initially, pearl millet flour samples were finely ground and thoroughly homogenised to ensure efficient lipid extraction. The lipid fraction was then quantitatively extracted from 5 g of the prepared flour using a Soxhlet apparatus with petroleum ether (boiling range 40–60 °C) as the solvent, continuing for 6–8 h until the solvent in the siphoning tube ran clear. Following extraction, the solvent was carefully removed from the lipid residue via rotary evaporation and subsequent drying in an oven at 100–105 °C for 30 min, after which the extracted fat was accurately weighed. For FFA determination, 1 g of the extracted lipid was dissolved in 50 mL of neutralised 95% ethanol, to which two to three drops of phenolphthalein indicator solution were added. The mixture was then titrated with a standardised 0.1 M potassium hydroxide (KOH) solution until a faint pink colour persisted for 30 s. A blank titration, conducted with neutralised ethanol and indicator but no lipid sample, was performed to correct for any acidity in the reagents. The FFA content, expressed as a % of oleic acid equivalents, was calculated based on the net volume of standardised KOH consumed, taking into account the molarity of the titrant and the equivalent weight of oleic acid.

### Enzymatic involvement in manipulating metabolites linked with off-odour in flour

#### Lipase activity assay

Lipase activity in pearl millet flour, a key factor in hydrolytic rancidity, was assessed spectrophotometrically by quantifying the release of *p*-nitrophenol from *p*-nitrophenyl butyrate (*p*-NPB), a synthetic substrate (18). For enzyme extraction, 0.5 g of homogenised flour was combined with 5 mL of ice-cold enzyme extraction buffer (100 mM sodium phosphate buffer, pH 7.5, containing 0.5% v/v Triton X-100) and vigorously vortexed for 1 min, followed by agitation on an orbital shaker at 180 rpm for 30 min at 4 °C. The suspension was then centrifuged at 10,000 × *g* for 15 min at 4 °C, and the resulting supernatant, containing the crude enzyme extract, was kept on ice for immediate assay. The lipase assay was performed by adding 100 μL of the enzyme extract to 900 μL of a 1 mM *p*-NPB assay solution (prepared by diluting a 50 mM *p*-NPB stock solution in acetonitrile into 100 mM sodium phosphate buffer, pH 7.5). The reaction mixture was incubated at 37 °C, and the increase in absorbance at 405 nm (A405) was continuously monitored over 10 min using a microplate reader. A blank reaction, substituting enzyme extract with extraction buffer, was run simultaneously to account for spontaneous substrate hydrolysis. A standard curve of *p*-nitrophenol was generated to convert absorbance changes into micromoles of *p*-nitrophenol liberated.

### Determination of lipoxygenase activity in flour

Lipoxygenase (LOX) activity in pearl millet flour, a significant contributor to oxidative rancidity, was determined spectrophotometrically by monitoring the formation of conjugated dienes at 234 nm, based on a modified method of Surrey, (19). For enzyme extraction, 1 g of finely powdered, homogenised pearl millet flour was vigorously mixed with 10 mL of ice-cold 0.2 M sodium phosphate buffer (pH 6.5) in a 15 mL centrifuge tube. The mixture was then centrifuged at 10,000 × *g* for 30 min at 4 °C, and the resulting supernatant, containing the crude LOX enzyme extract, was kept on ice for immediate assay. The LOX assay was performed in a 3 mL quartz cuvette at 25 °C. The reaction mixture consisted of 2.575 mL of 0.2 M sodium phosphate buffer (pH 6.5) and 60 μL of a freshly prepared linoleic acid substrate solution. The reaction was initiated by adding 250 μL of the crude enzyme extract, and the increase in absorbance at 234 nm was immediately recorded for 5 min in kinetic mode using a spectrophotometer. A blank reaction, replacing the enzyme extract with extraction buffer, was run to correct for non-enzymatic oxidation. LOX activity was calculated from the linear portion of the absorbance curve, using the molar extinction coefficient of 25,000 M^−1^cm^−1^ for conjugated diene hydroperoxides. The protein estimation was carried out using the method of Bradford (20).

### Peroxidase (POX) activity assay

Peroxidase (POX) activity in pearl millet flour was determined spectrophotometrically by monitoring the oxidation of guaiacol in the presence of hydrogen peroxide, following a modified method (21). For enzyme extraction, 1.0 g of homogenised flour was mixed with 5 mL of ice-cold 100 mM sodium phosphate buffer (pH 6.0), vortexed for 30 s and agitated on an orbital shaker at 150 rpm for 20 min at 4 °C. The suspension was then centrifuged at 10,000 × *g* for 10 min at 4 °C, and the resulting supernatant, containing the crude peroxidase, was kept on ice. The POX assay was carried out in a 3 mL quartz cuvette at 25 °C. The reaction mixture comprised 2.7 mL of 100 mM sodium phosphate buffer (pH 6.0), 100 μL of 20 mM guaiacol solution, and 100 μL of 5 mM H₂O₂ solution. The reaction was initiated by adding 100 μL of the enzyme extract, and the increase in absorbance at 470 nm was immediately recorded for 5 min in kinetic mode. A blank reaction, substituting enzyme extract with extraction buffer, was performed to account for non-enzymatic oxidation. POX activity was calculated from the linear portion of the absorbance curve using the molar extinction coefficient of 26.6 mM^−1^cm^−1^ for tetraguaiacol.

### Determination of polyphenol oxidase (PPO) activity

Polyphenol oxidase (PPO) activity in pearl millet flour was determined spectrophotometrically by monitoring the oxidation of catechol to quinones, absorbing at 420 nm, following a modified method (22). For enzyme extraction, 1.0 g of finely powdered, homogenised flour was ground with 5 mL of ice-cold enzyme extraction buffer (50 mM sodium phosphate buffer, pH 6.5, containing 1% w/v polyvinylpolypyrrolidone and 0.5% v/v Triton X-100) in a pre-chilled mortar. The resulting slurry was transferred to a centrifuge tube and centrifuged at 12,000 × *g* for 20 min at 4 °C. The supernatant, containing the crude PPO enzyme, was collected and kept on ice for immediate assay. The PPO assay was carried out in a 3 mL quartz cuvette at 30 °C. The reaction mixture comprised 2.7 mL of 50 mM sodium phosphate buffer (pH 6.5) and 100 μL of 100 mM catechol substrate solution (prepared fresh and kept on ice). The reaction was initiated by adding 200 μL of the enzyme extract, and the increase in absorbance at 420 nm was immediately recorded for 5 min in kinetic mode. A blank reaction, substituting enzyme extract with extraction buffer, was performed to correct for non-enzymatic oxidation. The total soluble protein was estimated using the method of Bradford (20).

### Statistical analysis

All experiments included three biological replicates with assays in triplicate. Results for acid value, peroxide value, and enzyme activities were expressed as mean ± SD. Statistical differences were analysed using one-way ANOVA and Tukey’s HSD (*p* < 0.05) in R software (version 4.5.1) with appropriate packages.

## Results and discussion

Low shelf-life is one of the major constraints in pearl millet processing, product development, consumption, and commercialisation. Although, several technologies and research efforts have targeted this issue, success has been limited. This is primarily due to limited understanding of the mechanisms underlying lipid oxidation and the involvement of enzymes and metabolites in the development of off-odours and rancidity in pearl millet flour (5, 23). The current study aimed to identify the key metabolites which could provide a biochemical baseline to better understand the rancidity development in hybrid of pearl millet.

### Metabolites signature in fresh flour of pearl millet

#### Metabolites in fresh flour of landrace (Chadhi Bajri)

We have identified potential metabolites in the fresh flour of Chadhi Bajri based on average peak area and observed a diverse biochemical profile, with significant representation from fatty acids, amino acids, organic acids, aldehydes, hydroperoxides and secondary metabolites (Supplementary Table S1). These metabolites not only contribute to the nutritional profile of the flour but may also play roles in resisting or delaying the onset of rancidity, making them valuable reference points for comparative studies with other genotypes of pearl millet.

Among the most abundant metabolites were asparagine, serine, and threonine, indicating a high level of free amino acids (FAAs), which may be linked to better protein turnover or stress response pathways. The findings are in conformity with the observation of (24). These amino acids have been associated with antioxidant roles and amino acid-derived signalling that may protect against oxidative stress (25). The presence of D-Glucono-1, 5-lactone 6-phosphate, an intermediate in the pentose phosphate pathway, suggests active sugar catabolism or antioxidant potential via NADPH production, which supports redox balance (26). S-Adenosylmethioninamine, a precursor in polyamine synthesis, is often involved in stress signalling and may contribute to cellular protection against lipid peroxidation.

Importantly, several lipid-derived compounds such as palmitic acid, oleic acid, and stearic acid were also detected in higher amount. These fatty acids are key components of lipid metabolism and are prone to oxidation during storage, making them critical markers for assessing rancidity onset and progression (27). Secondary metabolites such as phytol (a chlorophyll-derived alcohol with antioxidant properties) and pelargonidin derivatives (anthocyanins) are known for their radical scavenging ability and may delay the onset of oxidative rancidity (28). The detection of acetamide, malonyl-ACP, and (R)-S-lactoylglutathione further points to metabolic activities associated with nitrogen metabolism, fatty acid biosynthesis, and detoxification pathways, suggesting a robust biochemical defence system in fresh flour of Chadhi Bajri. Comparative profiling with rancid samples will help pinpoint specific markers and pathways altered during deterioration, potentially guiding breeding and storage strategies to enhance the flour shelf life (29).

#### Metabolites in fresh flour of hybrid Pusa-1201

The analysis of fresh flour of Pusa-1201 reveals its most abundant metabolites, providing a baseline understanding of its inherent metabolic composition (Supplementary Table S2). The metabolites, identified by their average peak areas, primarily consist of compounds central to primary metabolism, reflecting the healthy physiological state of the fresh flour. Dominating the profile is glycerol, present at an exceptionally high abundance of 1.0 × 10^8^, indicating its significant role as a structural component in glycerolipids or a metabolic intermediate within the flour matrix. Other highly abundant metabolites include various sugars and sugar phosphates like inosine (9.4 × 10^6^), D-glucuronate (8.8 × 10^6^), D-fructose (8.4 × 10^6^), glucose 1-phosphate (7.7 × 10^6^), glucose (7.6 × 10^6^), D-glucose 6-phosphate (7.0 × 10^6^), and D-mannose (6.2 × 10^6^). These suggest a rich presence of carbohydrates and active carbohydrate metabolism within fresh flour, serving as primary energy reserves and structural components. These findings are consistent with recent work by Zhong et al. (30), who used widely targeted metabolomics to examine wheat flours and their responses to nitrogen application. Their study similarly identified sugars, amino acids, nucleotide derivatives, and lipid-associated compounds as key determinants of flour quality and metabolic health. Furthermore, key intermediates of the tricarboxylic acid cycle and amino acid metabolism, such as glutarate (8.6 × 10^6^), L-glutamate (8.0 × 10^6^), pyruvate (6.7 × 10^6^), L-aspartate (6.3 × 10^6^), and 2-oxoglutarate (6.0 × 10^6^), are also highly abundant. The high levels of riboflavin (8.6 × 10^6^) and adenosine (6.8 × 10^6^) indicate the presence of essential cofactors and building blocks for nucleic acids and energy currency. Collectively, this profile reflects the robust metabolic integrity of fresh Pusa-1201 flour, characterised by ample reserves of energy-related compounds, building blocks for macromolecules, and key metabolites involved in fundamental biochemical processes.

### Metabolites signature in rancid flour of pearl millet

#### Metabolites in rancid flour of Pusa-1201 (hybrid)

The metabolite analysis of rancid Pusa-1201 flour reveals major biochemical shifts associated with rancidification (Table 1; Supplementary Table S3). Glycerol, with a peak area of 1.2 × 10^8^, was the most abundant metabolite, likely due to the breakdown of glycerolipids during storage. Other key metabolites—ethanolamine (1.5 × 10^7^) and D-glucono-1,5-lactone 6-phosphate (1.3 × 10^7^)—highlight active phospholipid and carbohydrate degradation. High levels of nucleotide derivatives such as guanine, UTP, and 2,5-diamino-6-(5-phospho-D-ribosylamino)pyrimidin-4(3H)-one indicate significant nucleic acid breakdown and disruption of energy metabolism. Studies show that oxidative stress (common in rancidity/lipid peroxidation) generates reactive oxygen species (ROS) which oxidise purine and pyrimidine bases, producing oxidised derivatives of guanine (e.g., 8-oxoG), and damage nucleic acids. This breakdown can increase free guanine or modified guanine nucleotides in metabolomic profiles (31). Additionally, lipid and pigment degradation products like pregnenolone and aniline were detected in large amounts, pointing to extensive oxidative stress and complex chemical transformations. These metabolite accumulations collectively reflect the degradative processes triggered by storage, compromising flour quality and stability. This profile underscores the impact of rancidity on Pusa-1201 flour and highlights key biochemical markers useful for monitoring spoilage and improving post-harvest management strategies in pearl millet. The findings are in conformity with the observation of Zhu et al. (32) who reported a diverse array of sugars, sugar phosphates, amino acids, TCA intermediates, and lipid-related metabolites across different grain layers in wheat.

**Table 1:** Top 20 metabolites identified in rancid flour of Pusa-1201, ranked by average peak area.

Metabolites	Molecular formula	Average peak area
Glycerol	C_3_H_8_O_3_	1.20 × 10^8^
Ethanolamine	C_2_H_7_NO	1.50 × 10^7^
D-Glucono-1,5-lactone 6-phosphate	C_6_H_11_O_9_P	1.30 × 10^7^
D-Fructose	C_6_H_12_O_6_	9.80 × 10^6^
Guanine	C_5_H_5_N_5_O	8.40 × 10^6^
2,5-Diamino-6-(5-phospho-D-ribosylamino)pyrimidin-4(3H)-one	C_9_H_16_N_5_O_8_P	8.30 × 10^6^
chlorophyllide b	C_35_H_32_MgN_4_O_6_	8.40 × 10^6^
Dihydrozeatin	C_10_H_15_N_5_O	7.90 × 10^6^
Tyramine	C_8_H_11_NO	6.90 × 10^6^
Lariciresinol	C_20_H_24_O_6_	6.90 × 10^6^
UTP	C_9_H_15_N_2_O_15_P_3_	6.00 × 10^6^
Sucrose	C_12_H_22_O_11_	4.60 × 10^6^
Pregnenolone	C_21_H_32_O_2_	4.30 × 10^6^
Aniline	C_6_H_7_N	4.20 × 10^6^
17-O-Acetylajmaline	C_22_H_28_N_2_O_3_	4.10 × 10^6^
Deoxycytidine	C_9_H_13_N_3_O_4_	3.90 × 10^6^
Bergapten	C_12_H_8_O_4_	3.90 × 10^6^
trans-Oct-2-enoyl-[acp]	C_8_H_13_OSR	3.70 × 10^6^
5-Amino-6-(5′-phosphoribosylamino)uracil	C_9_H_15_N_4_O_9_P	3.70 × 10^6^
Salicylate	C_7_H_6_O_3_	3.60 × 10^6^
Glutamine	C_5_H_10_N_2_O_3_	4.10 × 10^6^
cis-Aconitate	C_6_H_6_O_6_	3.60 × 10^6^
D-Glucose 6-phosphate	C_6_H_13_O_9_P	3.50 × 10^6^
Guanosine	C_10_H_13_N_5_O_5_	3.50 × 10^6^
Aminoimidazole ribotide	C_8_H_14_N_3_O_7_P	3.50 × 10^6^

### Distribution of metabolites in fresh and rancid flours of Chadhi Bajri and Pusa-1201

**Figure 1:**
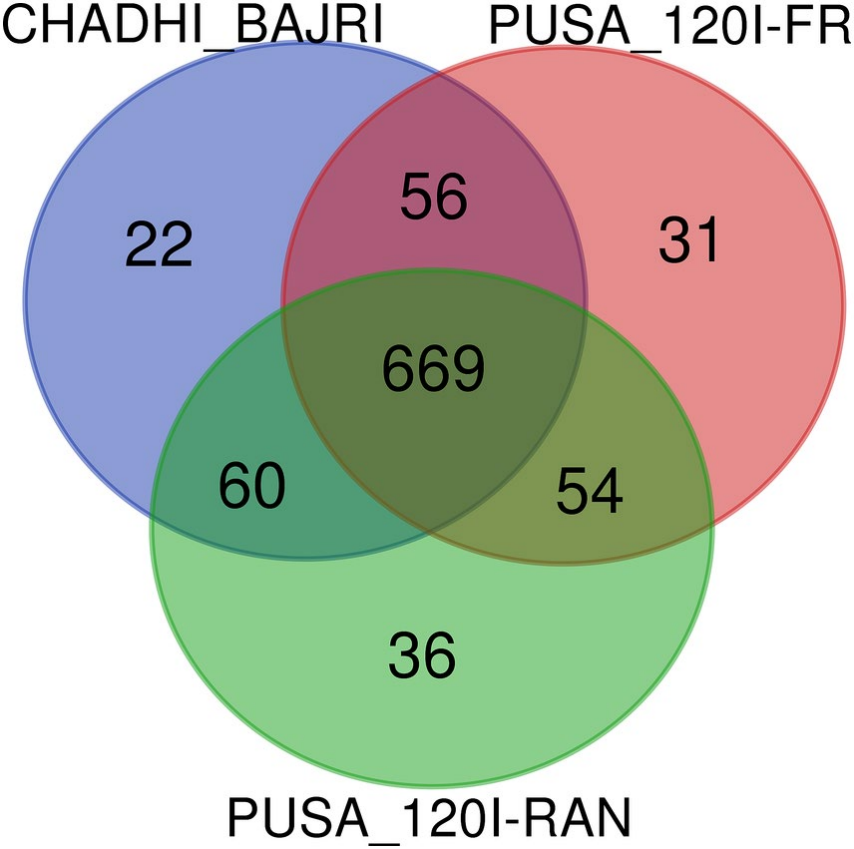
Venn diagram showing the distribution of detected metabolites (unique and differentially expressed) in fresh and rancid flours of pearl millet cultivars, Pusa-1201 and Chadhi Bajri. Chadhi Bajri—fresh flour, PUSA_1201-FR—fresh flour, PUSA_1201-RAN—rancid flour.

The Venn diagram analysis illustrates the metabolite distribution among Chadhi Bajri (fresh), Pusa-1201 (fresh), and Pusa-1201 (rancid) flours (Figure 1). A core set of 669 metabolites was shared across all samples, indicating a conserved baseline metabolic profile. Chadhi Bajri exhibited 22 unique metabolites, suggesting cultivar-specific traits potentially linked to better oxidative stability. Pusa-1201 fresh and rancid flours showed 31 and 36 unique metabolites, respectively, with the latter likely associated with rancidity-induced degradation. Additionally, 56 metabolites were common between the fresh flours of both cultivars, and 60 were shared between Chadhi Bajri and rancid Pusa-1201, indicating overlap even under stress. The 54 shared metabolites between fresh and rancid Pusa-1201 reflect some metabolic continuity during spoilage. These findings highlight the metabolic distinctions between cultivars and the biochemical alterations associated with the development of off-odour and rancidity in pearl millet flour.

### Metabolomic variations between the fresh flours of Pusa-1201 and Chadhi Bajri of pearl millet

The volcano plot analysis comparing the fresh flour of Pusa-1201 (hybrid) and Chadhi Bajri (Landrace) revealed significant differences in their metabolic profiles, providing insights into potential metabolites linked with rancidity (Supplementary Figure S1a). The analysis identified several metabolites that were differentially abundant based on their log_2_ fold change (FC) and statistical significance (*p*-value < 0.05; Supplementary Table S4). Notably, metabolites upregulated in flour of Pusa-1201 included pregnenolone, quercetin 3-sulfate, gibberellin A24, (9Z)-hexadecenoic acid, malonyl-CoA, indole-5,6-quinone, 5-dehydroepisterol, and isopentenyl diphosphate. Among these, the significantly higher levels of (9Z)-hexadecenoic acid, an unsaturated fatty acid, in Pusa-1201 is important, as unsaturated fatty acids are highly prone to lipid oxidation, a primary driver of rancidity. PUFA are highly sensitive to autoxidation during flour storage, and forms hydroperoxides, aldehydes and ketones causes off-flavour formation (33, 34). This suggests a higher intrinsic potential for oxidative degradation in Pusa-1201. Conversely, metabolites that were downregulated in Pusa-1201 included dhurrin, desulfoglucotropaeolin, betaine aldehyde, (6S)-6-Hydroxy-1,4,5,6-tetrahydronicotinamide-adenine dinucleotide, rosmarinate, gibberellin A8, 7,9,9-tris-neurosporene, biliverdin, (R)-prunasin, 3-(3,4-Dihydroxyphenyl)pyruvate, bergapten, and 6-pyruvoyltetrahydropterin. The elevated levels of rosmarinate, a known antioxidant, in Chadhi Bajri, as compared to Pusa-1201, suggest that the landrace may possess a more robust inherent defence system against oxidative stress, contributing to potentially greater oxidative stability and reduced rancidity (35). Taken together, the findings support the hypothesis that Pusa-1201 flour, despite its agronomic advantages, may be biochemically predisposed to oxidative rancidity due to a higher content of oxidation-prone lipids and lower antioxidant metabolite reserves, whereas Chadhi Bajri demonstrates a metabolomic profile that favours oxidative stability and longer shelf life.

### Metabolomic variations between the rancid (Pusa-1201) and fresh flour (Chadhi Bajri) of pearl millet

The volcano plot presents a comparative analysis of metabolite profiles between the rancid flour of Pusa-1201 and fresh flour of Chadhi Bajri (used as reference material), aiming to identify metabolites associated with rancidity (Supplementary Figure S1b; Supplementary Table S5). Metabolites significantly more abundant in rancid flour of Pusa-1201 (down-regulated in Chadhi Bajri) include Betaine aldehyde, Red chlorophyll catabolite, Xanthonine, 3-Oxotetradecanoyl-CoA, Cortisol, bergapten, Glutamine 6-pyruvoyltetrahydropterin, Shikimate 3-phosphate, (E)-4-Hydroxyphenylacetaldehyde-oxime, Trans-Cinnamate, (S)-(+)-Allantoin, Biliverdin, and D-Ribose, among others. The presence of these metabolites in higher concentrations in rancid flour of Pusa-1201 could indicate different pathways of degradation or defence mechanisms at play in the hybrid variety. For instance, the differing levels of fatty acid derivatives (e.g., 3-Oxohexanoyl-CoA) could reflect distinct lipid degradation pathways and, consequently, varying profiles of rancid off-flavours between the two flours. Sravani et al. (36) performed comparative metabolomics on contrasting pearl millet genotypes to reveal how lipid catabolism drives rancidity and reported significantly elevated levels of fatty aldehydes (e.g., heptadecanal), fatty amides, and oxidised phospholipids and glycerolipids—consistent with our observations of elevated fatty acid derivatives in rancid flour of Pusa-1201. The analysis of fresh flours revealed several metabolites significantly elevated in Chadhi Bajri. These include Threonine, Quercetin 3-sulfate, 4-Coumaroylshikimate, Beta-Sitosterol, N-(L-Arginino)succinate, All-trans-Hexaprenyl diphosphate, 4-Oxo-1-(3-pyridyl)-1-butanone, 17-(2,2-Dimethyl-4-dodecateracnoyl-CoA), and others. The higher abundance of quercetin 3-sulfate in fresh flour of Chadhi Bajri might suggest a compensatory antioxidant response to ongoing oxidative stress, or it could be a metabolite of an antioxidant that has been consumed. Tiozon et al. (63) used untargeted etabolomics across cereal grains (including millet and sorghum) to map phenolics and flavonoids. They emphasised that fluctuations in flavonoid compounds (like quercetin derivatives) are indicative of dynamic antioxidant responses to oxidative stress. This aligns well with our finding of elevated quercetin 3-sulfate in fresh Chadhi Bajri flour, suggesting an active antioxidant defence. These findings highlight key metabolic differences between the rancid flour of Pusa-1201 (hybrid) and fresh flour of Chadhi Bajri (landrace), providing potential markers and insights into the biochemical processes contributing to rancidity in each flour type.

### Dynamic metabolic changes associated with the rancidification process in Pusa-1201

A comparative analysis of metabolites was carried out in rancid and fresh flour of Pusa-1201 (hybrid) to understand the mechanism underlying rancidification in flour (Supplementary Figure S1c; Supplementary Table S6). In this comparison, metabolites significantly up-regulated in rancid flour of Pusa-1201 (red points on the right side) include Leukotriene A4, 1,3-Diaminopropane, N-(L-Arginino)succinate, Indolylmethyl-desulfoglucosinolate, Methyl-2-O-(alpha-L-fucopyranosyl)-beta-D-galactoside, divinyl protochlorophyllide a, 7-hydroxy-chlorophyllide a, (R)-4-Hydroxy(phenyl)lactate, and 7-Dehydrodesmosterol. The presence of Leukotriene A4, a lipid-derived mediator often associated with oxidative stress and inflammation, in higher abundance in rancid flour strongly suggests its involvement in the oxidative degradation pathways leading to rancidity. The increase in other compounds could indicate the breakdown of larger molecules or the formation of new compounds as a result of the rancidification process.

On the contrary, metabolites significantly down-regulated in rancid flour of Pusa-1201 (blue points on the left side) include 2,5-Diamino-6-(5-phospho-D-ribosylamino)pyrimidin-4(3H)-one, Porphobilinogen, Guanosine, UTP, Dihydrozeatin-O-glucoside, (3R)-3-Hydroxybutanoyl-[acyl-carrier protein], DTTP, Chlorophyllide b, C25-Allenic-apo-aldehyde, DADP, Aminoimidazole ribotide, Citrate, Biliverdin, (9Z,11E)-(13S)-13-Hydroperoxyoctadeca-9,11-dienoic acid, Indole-5,6-quinone, Anandamide, Octane, and Pyruvate oxime. The decrease in certain nucleotide derivatives (UTP, Guanosine, DTTP) could reflect metabolic shifts or degradation of cellular components during rancidification. Notably, the significant decrease in (9Z,11E)-(13S)-13-Hydroperoxyoctadeca-9,11-dienoic acid, a lipid hydroperoxide, from fresh to rancid flour might indicate its consumption or further transformation into other rancidity-related compounds. The reduction in compounds like Chlorophyllide b and C25-Allenic-apo-aldehyde suggests the breakdown of chlorophylls and related pigments, which can also influence the colour and flavour of the flour as rancidity progresses. A non-targeted LC-HRMS metabolomic study of pearl millet flour demonstrated that rancidity is driven by increased lipid catabolism. This study reported elevated levels of fatty acid breakdown products, including fatty acyls, fatty aldehydes, and fatty amides (such as anandamide), in highly rancid samples (29).

**Figure 2:**
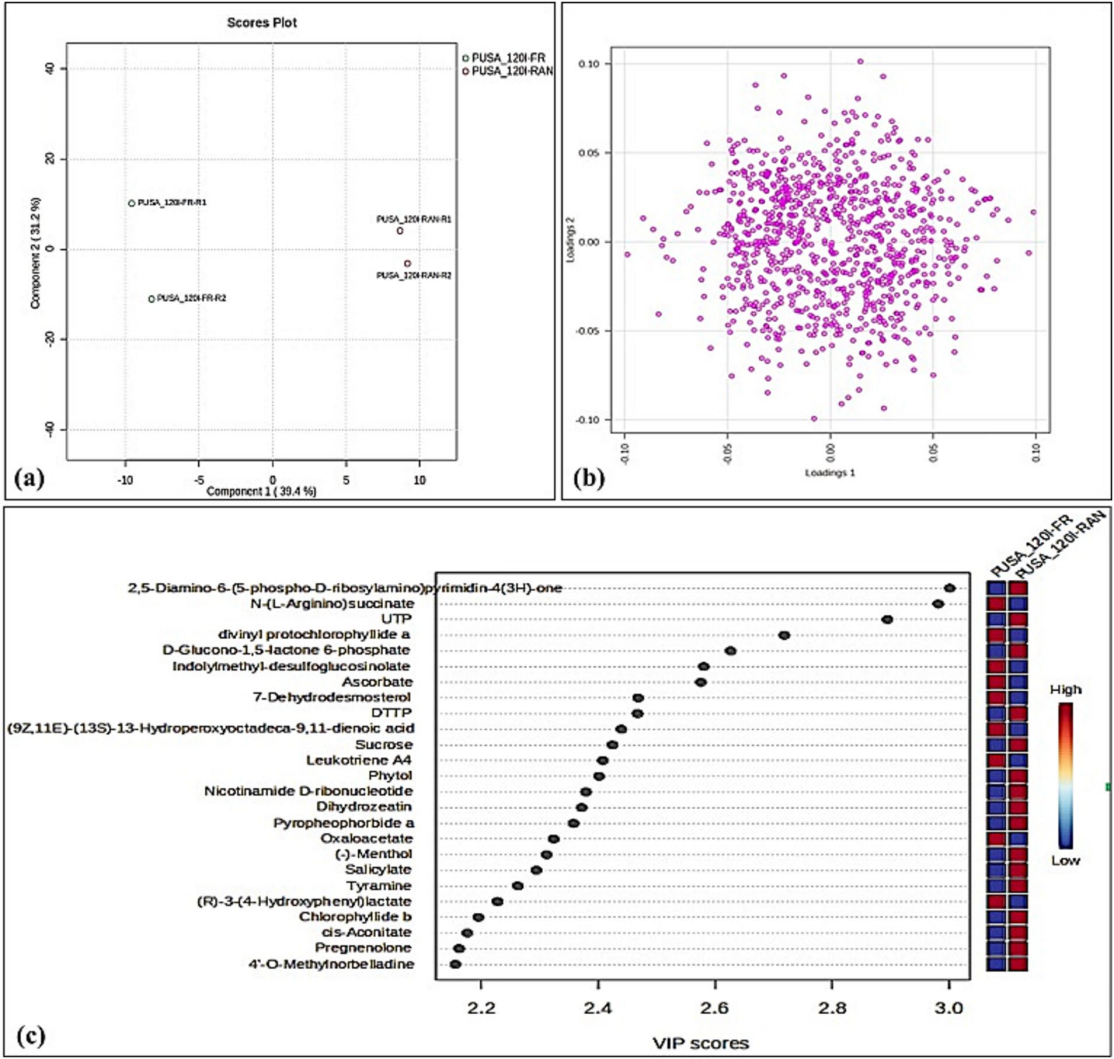
Multivariate analysis and identification of key discriminatory metabolites in fresh and rancid flour of Pusa-1201. **(a)** PLS-DA score plot showing clear separation between fresh (PUSA_1201-FR) and rancid (PUSA_1201-RAN) flour samples based on their metabolic profiles. **(b)** PLS-DA loading plot representing the contribution of each metabolite to the separation between sample groups. **(c)** VIP (variable importance in projection) score plot highlighting the top 25 metabolites contributing most to the group discrimination.

The PLS-DA scores plot reveals a clear metabolic separation between fresh and rancid Pusa-1201 flour, with Component 1 explaining 39.4% of the variance (Figure 2a). The close clustering of replicates demonstrates high reproducibility of metabolic changes during rancidification. The loadings plot indicates that a diverse set of metabolites drives this separation, with those having high positive loadings correlating with rancid flour and negative loadings with fresh flour (Figure 2b). This confirms a profound metabolic shift during rancidity, although specific metabolite identities need further investigation for a complete biochemical understanding of the process.

### Identification of potential metabolites markers linked with rancidification using PLS-DA variable importance in projection (VIP) plot analysis

The partial least squares-discriminant analysis (PLS-DA) variable importance in projection (VIP) plot served to identify the key metabolites driving the differentiation between fresh and rancid flour of Pusa-1201, with all listed metabolites exhibiting VIP scores well above the common threshold of 1.0, signifying their high discriminatory power (Figure 2c). Among the most influential metabolites, several were found to be notably depleted in rancid flour compared to fresh, including 2,5-Diamino-6-(5-phospho-D-ribosylamino)pyrimidin-4(3H)-one, UTP, DTTP, and particularly ascorbate, whose significant decrease underscores the consumption of critical antioxidants during the rancidification process. Ba et al. (37) analysed quinoa flour lipid oxidation during storage by UPLC-Orbitrap, applying VIP threshold (>1), identifying oxidised lipid species (e.g., TGs, DGs, FAs) as primary discriminators. Rancid flour showed increased metabolites like D-Glucono-1,5-lactone 6-phosphate and Leukotriene A4, confirming oxidative stress. The accumulation of phytol and divinyl protochlorophyllide further indicated chlorophyll degradation, providing strong indicators of metabolic shifts and oxidative degradation. Mach, (38) demonstrated that phytol—a breakdown product of chlorophyll—is released during chlorophyll catabolism and can be further channelled into tocopherol (vitamin E) biosynthesis, serving as an endogenous antioxidant in plants. Collectively, these highly discriminatory metabolites, reflecting changes in nucleotide metabolism, antioxidant status, and lipid degradation pathways, provide robust biomarkers for monitoring and understanding the intricate biochemical changes associated with rancidity development in Pusa-1201 flour.

### Heat-map to visualise the abundance of top 25 metabolites involved in flour rancidification

**Figure 3:**
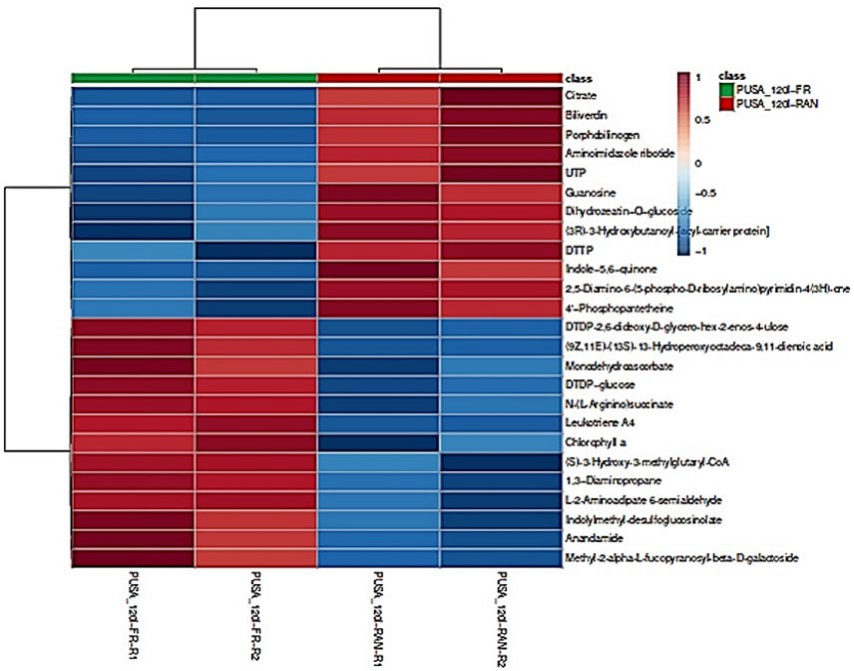
Heat-map of differentially expressed metabolites (DEMs) in fresh and rancid flour of Pusa-1201. The heat-map displays the relative abundance of 25 key metabolites in fresh (PUSA_1201-FR) and rancid (PUSA_1201-RAN) flour samples. Metabolite abundance is represented by colour intensity, with red indicating higher levels and blue indicating lower levels.

The heat-map visually illustrates the relative abundance of 25 key metabolites in fresh (PUSA_1201-FR) and rancid (PUSA_1201-RAN) flour of Pusa-1201, revealing distinct metabolic shifts associated with rancidification (Figure 3). The samples cluster into two well-defined groups, confirming that rancidity significantly alters the flour’s metabolic profile. Two primary metabolite clusters with opposing abundance patterns emerge.

The first cluster includes metabolites more abundant in fresh flour—represented in red in fresh samples and blue in rancid ones. These include energy-related compounds and antioxidants such as UTP, Guanosine, DTTP, Citrate, and Indole-5,6-quinone. Lipid hydroperoxide ((9Z,11E)-(13S)-13-Hydroperoxyoctadeca-9,11-dienoic acid), a marker of primary oxidation, is also notably reduced in rancid samples, indicating progression to secondary oxidative products (aldehydes, ketones). These changes suggest consumption of energy reserves and antioxidants, reflecting oxidative stress and compromised cellular integrity during storage. Lipid hydroperoxides, initially formed through autoxidation, are rapidly degraded via radical or metal-catalysed cleavage into secondary products (e.g., aldehydes, ketones), which are responsible for off-flavours and deterioration during storage (39).

The second cluster is enriched in rancid flour samples, including Leukotriene A4, DTDP-glucose, Chlorophyll a, N-(L-Arginino)succinate, and 1,3-Diaminopropane. The sharp increase in Leukotriene A4 confirms active lipid peroxidation, while elevated levels of pigment-related compounds (e.g., Chlorophyll a) imply pigment breakdown. These alterations align with oxidative damage and biochemical degradation that drive off-flavour formation and colour changes in spoiled flour.

Overall, the heat-map validates significant and reproducible metabolic divergence between fresh and rancid states, identifying metabolite signatures that serve as indicators of oxidative and hydrolytic spoilage. These findings reinforce the mechanistic basis for rancidity progression and offer valuable targets for monitoring and mitigating flour deterioration in pearl millet. Sonar et al. (40) highlight metabolic shifts affecting pigment composition that contribute to colour changes and quality loss during storage and oxidative stress, consistent with our findings in rancid flour.

### Mapping networks and pathways of potential metabolite markers linked with rancidity

#### Lipid metabolism pathway

Based on the metabolic analysis, differences in lipid metabolism pathway, particularly those related to phytosterol and jasmonic acid biosynthesis, influence rancidity susceptibility in flours (Supplementary Figure S2a). Chadhi Bajri showed maximum accumulation of pro-oxidant (9Z)-Hexadecenoic acid and Malonyl-CoA, suggesting susceptibility to lipid peroxidation. It also has higher 5-Dehydroepisterol. In contrast, Pusa-1201 exhibited maximum levels of the antioxidant *β*-Sitosterol, which protects against oxidative degradation and stabilises membranes. These inherent variations in lipid composition and metabolism explain the distinct rancidity profiles of the two flours. Yoshida and Niki (41) demonstrated that β-sitosterol effectively suppresses lipid oxidation and protects tocopherol, acting both as a free radical scavenger and membrane stabiliser. These findings collectively suggest that inherent differences in lipid composition and metabolism, particularly concerning fatty acid saturation and phytosterol content, play a crucial role in the distinct rancidity profiles observed between the flours of Chadhi Bajri (landrace) and Pusa-1201 (hybrid).

#### Secondary metabolites

The metabolic network map highlights the biosynthesis of various secondary metabolites, particularly focusing on flavonoids (M00137 Flavanone biosynthesis, M00138 Flavonoid biosynthesis) and monolignols (M00039) (Supplementary Figure S2b). These pathways are crucial as they produce compounds with known antioxidant capacities, directly impacting the oxidative stability and rancidity of flour. Consistent with this, our metabolomic analysis revealed differential abundance of Quercetin 3-sulfate, a flavonoid derivative, which was consistently found in higher levels in both Chadhi Bajri and Pusa-1201. Elevated levels of Quercetin 3-sulfate in both flours reflect active flavonoid production that enhances oxidative defence, aligning with the flavonoid biosynthesis pathway highlighted in the metabolic network (42). This suggests that the landrace may possess a higher intrinsic capacity for producing certain antioxidant secondary metabolites, potentially as a defence mechanism against oxidative stress. Flavonols such as quercetin glycosides exhibit strong antioxidant and antimicrobial activities, which help extend shelf life by inhibiting lipid oxidation in various foods (43). Both flours showed elevated quercetin 3-sulfate, indicating an intrinsic flavonoid-based antioxidant defence. While trans-cinnamate, a phenolic precursor, increased in rancid Pusa-1201 flour, a universal link to monolignol changes was not confirmed. These findings suggest that variations in flavonoid biosynthesis and other phytochemicals contribute to the distinct oxidative stability and rancidity profiles of the flours.

#### Terpenoid pathway

Based on the metabolic network map, C5 isoprenoid biosynthesis through mevalonate and non-mevalonate pathways are foundational for terpenoid production (Supplementary Figure S2c). Key terpenoid pathways highlighted include those for antioxidant beta-carotene, abscisic acid, and gibberellins. Beta-carotene’s presence can mitigate oxidative damage and its degradation may signal flour spoilage. Ekpa et al. (44) demonstrated that β-carotene and related carotenoids in biofortified maize flour undergo significant degradation during storage with retention dropping to 16–73% over 180 days in different packaging types. The marked decrease in β-carotene during storage confirms its antioxidant role initially, but also highlights its vulnerability to oxidative degradation. While the map focuses on synthesis, changes in the levels of these compounds or their precursors, as observed in metabolomic studies, could provide insights into the flour’s oxidative stability and the biochemical mechanisms underlying rancidity development. Rodriguez-Concepcion et al. (64) reviewed the mevalonate (MVA) and MEP/DOXP pathways as vital sources of isoprenoid precursors leading to carotenoids, abscisic acid (ABA), and gibberellins in plants. Mapping these central terpenoid pathways justifies the networking of β-carotene, ABA, and GA biosynthesis, and shifts in their metabolic flows influencing the oxidative resilience of flour—particularly during storage.

### Biochemical signatures of differentially expressed metabolites (DEMs) linked with rancidity

The analysis comparing fresh flour of Chadhi Bajri and rancid flour of Pusa-1201 reveals significant differences in metabolite profiles, providing deeper insights into varietal specificities of rancidity development. The observed high fold changes indicate that rancid flour of Pusa-1201 exhibits a more pronounced accumulation of specific compounds, suggesting distinct biochemical pathways or a greater extent of degradation in flour. Key categories of metabolites with substantial fold increases in rancid flour of pearl millet cv. Pusa-1201 includes the following.

### Lipid oxidation by-products

Several metabolites directly point to extensive lipid degradation, a hallmark of rancidity. Compounds like 3-Oxotetradecanoyl-CoA (28.10-fold increase) and 3-Oxooctanoyl-[acp] (17.85-fold increase) are acyl-CoA/ACP derivatives, indicating active fatty acid breakdown pathways. The presence of (9S,10S)-9,10-Dihydroxyoctadecanoate (15.54-fold increase) and Leukotriene A4 (15.03-fold increase) further suggests specific enzymatic and non-enzymatic oxidation routes of unsaturated fatty acids. The elevated C25-Allenic-apo-aldehyde (13.11-fold increase) points to the formation of volatile aldehydes, which are primary contributors to undesirable rancid off-flavours. Chang et al. (45) reported that secondary oxidation products such as dihydroxy fatty acids (like 9,10-dihydroxyoctadecanoate), hydroperoxides, and volatile aldehydes are synthesised via enzymatic and non-enzymatic oxidation pathways and recommended volatile aldehydes as key markers of rancidity due to their strong sensory impact.

### Nucleic acid and energy metabolism indicators

Metabolites such as Xanthosine (92.80-fold increase), Deoxycytidine (34.63-fold increase), and Guanosine-3′-diphosphate-5′-triphosphate (19.93-fold increase) show the highest fold changes. These profound increases suggest significant degradation of nucleic acids (DNA/RNA) and disruptions in nucleotide metabolism, which could be a consequence of cellular damage and general metabolic collapse occurring during severe rancidification. Pan et al. (46) reported that nucleotides and nucleotide derivatives (such as AMP, IMP, inosine, hypoxanthine, xanthine) undergo dramatic turnover during spoilage, often leading to accumulation of degradation products like xanthine and nucleosides. This reflects extensive nucleotide breakdown linked to tissue deterioration and enzymatic activity during spoilage.

### Pigment and secondary metabolite degradation

The elevated levels of red chlorophyll catabolite (18.33-fold increase), chlorophyllide b (14.52-fold increase), and [PR] cis-Phytoene (13.17-fold increase) highlight the breakdown of photosynthetic pigments. This degradation can contribute to discoloration and indicates a broader oxidative environment within the flour matrix. Additionally, compounds like DIMBOA (48.39-fold increase), Lariciresinol (36.30-fold increase), Bergapten (25.07-fold increase), and Sanguinarine (21.07-fold increase), often associated with plant defence mechanisms, show significant changes. Their altered levels may reflect the plant’s response to oxidative stress or their own chemical transformation under rancid conditions. The elevated levels of DIMBOA, lariciresinol, bergapten, and sanguinarine in rancid flour likely reflect an oxidative stress–induced chemical response or transformation of defence pathways. Benzoxazinoids like DIMBOA are well-recognised stress-responsive secondary metabolites in cereals such as maize, wheat, and rye. These compounds accumulate in response to oxidative or biotic stress and are most concentrated in grain components like the germ and bran (47). The present findings demonstrate that pigment catabolism and stress-induced benzoxazinoid accumulation are valid biochemical indicators of oxidative deterioration and rancidity in pearl millet flour matrices.

### Pathways responsible for rancidification in Pusa-1201 (hybrid)

The potential metabolites identified exhibit substantial fold changes between fresh and rancid samples, with most showing a marked increase in concentration in the rancid state. These shifts highlight the biochemical pathways actively engaged during the spoilage of the flour. Several categories of metabolites demonstrate significant involvement.

### Carbohydrate and energy metabolism intermediates

Metabolites such as D-Glucono-1,5-lactone 6-phosphate (177.90-fold increase), Triphosphate (80.22-fold increase), UTP (63.99-fold increase), and Sucrose (20.48-fold increase) show considerable accumulation. This suggests a disruption in normal carbohydrate metabolism and energy homeostasis, possibly due to enzymatic degradation or non-enzymatic reactions that occur as the flour ages and deteriorates. The breakdown of complex carbohydrates into simpler sugars and phosphorylated intermediates can fuel microbial growth or participate in Maillard reactions, contributing to off-flavours and discoloration. Reducing sugars and phosphorylated carbohydrates (such as sugar phosphates) undergo non-enzymatic browning during storage, leading to melanoidins, volatile flavour compounds (e.g., aldehydes), discoloration, and nutrient loss. These reactions explain the accumulation of sugar and phosphate metabolites—like D-Glucono-1,5-lactone-6-phosphate and sucrose—and their role in off-flavours and discoloration observed in aged flour (48).

### Lipid oxidation products and precursors

Compounds directly linked to lipid degradation, such as Phytol (28.89-fold increase), and fatty acid derivatives like trans-Oct-2-enoyl-[acp] (17.90-fold increase) and Hexadecanoic acid (3.55-fold increase), are highly elevated. Rancidity is primarily driven by lipid oxidation, where unsaturated fatty acids react with oxygen, forming hydroperoxides that then break down into various volatile aldehydes, ketones, and carboxylic acids responsible for characteristic rancid odours. The increase in these lipid-related compounds indicates active oxidative degradation of lipids within the flour. Pathways like autoxidation, photo-oxidation, and enzymatic processes causes’ lipid oxidation. Among these pathways, autoxidation is the most potent pathways generating hydroperoxides and other compounds like aldehydes and ketones. Most of these compounds are volatile and responsible for off-flavours in oxidised food products (49).

### Amino acid and protein degradation products

Ethanolamine (22.90-fold increase) and Tyramine (12.11-fold increase), along with changes in amino acids like Lysine (3.87-fold increase) and Glutamine (3.00-fold increase), point towards protein and phospholipid degradation. The breakdown of proteins and amino acids can lead to the formation of amines and other nitrogenous compounds, which contribute to undesirable flavours and odours in aged flour. Ruiz-Capillas and Herrero (50) reported the biochemical origins of amines such as ethanolamine and tyramine from protein and phospholipid breakdown and highlighted their sensory effects. Shahidi and Zhong (51) elucidated the degradation pathways of lipid and phospholipid involved in the formation of off-flavour and rancidity.

### Pigment degradation markers

Chlorophyllide b (12.56-fold increase) and Pyropheophorbide a (14.83-fold increase) are significant indicators. These are breakdown products of chlorophyll, suggesting oxidative degradation of pigments, which can lead to colour changes in the flour and potentially contribute to the overall oxidative stress within the matrix. The accumulation of these catabolites suggests that oxidative stress in the flour matrix triggers pigment breakdown pathways similar to those observed during leaf senescence and plant tissue aging (52). This pigment degradation not only contributes to the discoloration characteristic of rancid flour but also reflects the broader oxidative environment affecting lipids, proteins, and other biomolecules. Hence, chlorophyll catabolites serve as important biochemical markers for the extent of oxidative spoilage and may also influence the sensory quality of the flour through changes in colour and potential secondary reactions.

### Secondary metabolites and stress response compounds

Compounds like Salicylate (14.39-fold increase) and various steroids (Pregnenolone, Estradiol-17beta) also showed elevated levels. These may reflect either the plant’s natural stress response to deterioration or their own degradation pathways under oxidative conditions. Oxidative stress causes damage to the macro- and micronutrients (53). Plants do produce their own steroids, known as phytosteroids or brassinosteroids. These compounds are involved in growth, development, and stress responses. However, the presence of specific animal steroids like pregnenolone and estradiol-17beta is less common and may point to alternative metabolic processes (54).

The identified metabolites provide a comprehensive molecular signature of rancidity in the flour of Pusa-1201 (hybrid), indicating widespread degradation of carbohydrates, lipids, and proteins, alongside the breakdown of natural pigments. These changes collectively contribute to the deterioration of sensory qualities and overall shelf-life of the flour.

### Changes in lipid degradation indices in flours during storage

**Figure 4:**
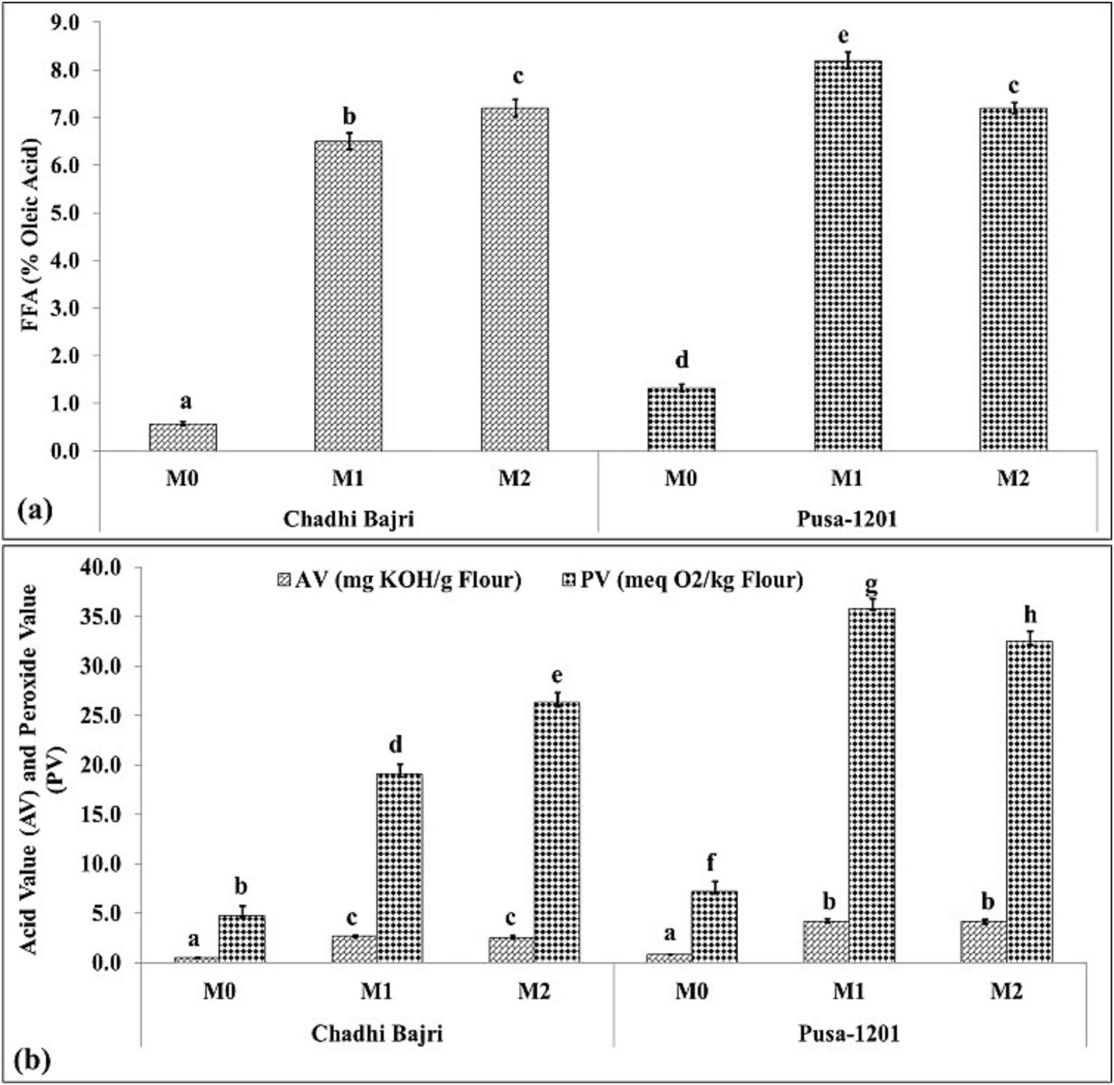
Progression of rancidity in Chadhi Bajri and Pusa-1201 flours stored over time, **(a)** acid value (AV) indicating hydrolytic rancidity, **(b)** peroxide value (PV), representing oxidative rancidity, **(c)** free fatty acid (FFA) content. A direct measure of lipid hydrolysis. Data are presented for fresh (M_0_), 1-month (M_1_), and 2-month (M_2_) stored samples. Values are expressed as mean ± standard deviation (*n* = 3). Statistical significance between groups at different time points is indicated by letters above histogram.

The changes in the lipid degradation indices like acid value (AV), peroxide value (PV) and free fatty acids (FFAs) were estimated in fresh (M_0_), 1-month stored (M_1_), and 2-month stored (M_2_) flour samples of Chadhi Bajri (landrace) and Pusa-1201 (hybrid) *cvs.* of pearl millet. In fresh flour (M_0_), both cultivars exhibited minimal FFAs levels, with Chadhi Bajri showing the minimum (0.6%) and Pusa-1201 slightly higher (1.4%) (Figure 4a). Upon storage, a marked increase in FFAs content was observed. In Chadhi Bajri, FFA increased significantly to 6.9% in <<>M_1_ and reached 7.3% in M_2_. In contrast, Pusa-1201 displayed a more drastic increase, with FFA peaking at 8.4% in M_1_ before slightly decreasing to 7.2% in M_2_. These results indicate pronounced lipolytic activity in both cultivars during storage, with Pusa-1201 being more susceptible to FFA accumulation and thus more prone to rancidity. Aher et al. (6) identified mutated lipase genes in specific pearl millet lines and observed decrease in the rancidity in flour. This finding provides compelling evidence for the role of lipase activity in free fatty acid (FFA) accumulation and explains the differential susceptibility of various cultivars to rancidity. Further, we analysed the changes in acid value (AV) and peroxide value (PV) which is used as indicators of hydrolytic and oxidative rancidity, respectively (Figure 4b). In Chadhi Bajri, AV increased modestly from M_0_ (3.0 mg KOH/g) to M_1_ (4.5 mg KOH/g) and remained similar in M_2_ (4.6 mg KOH/g). Correspondingly, PV values rose significantly from 6.5 meq O₂/kg (M_0_) to 19.4 meq O₂/kg (M_1_), and further to 27.5 meq O₂/kg (M_2_), indicating progressive lipid peroxidation. In Pusa-1201, the AV remained constant across all stages (~4.7 mg KOH/g), but PV increased dramatically from 5.9 (M_0_) to 36.7 (M_1_) and slightly reduced to 33.1 meq O₂/kg in M_2_. This sharp rise in PV highlights the oxidative instability of Pusa-1201 flour under storage, corroborating the elevated FFA levels observed in panel (a). (36) reported that the presence of both lipase and lipoxygenase enzymes in pearl millet leads to a rapid increase in both FFAs and peroxides. This directly supports our observation that both AV and PV increased, indicating the progression of both rancidity pathways.

Collectively, these findings suggest that both Chadhi Bajri and Pusa-1201 undergo significant lipid degradation during storage, with Pusa-1201 exhibiting a higher tendency towards oxidative rancidity. These biochemical changes underline the importance of cultivar selection and storage conditions in maintaining the shelf-life and nutritional quality of pearl millet. Goswami et al. (14) showed that CPV reaches its maximum value on the 10^th^ day of storage, after which it decreases due to the breakdown of hydroperoxides into aldehyde and other ketone products. This directly justifies our observation in Pusa-1201, where PV slightly reduced to 33.1 meq O₂/kg in M_2_ after peaking in M_1_. This finding demonstrates that a decrease in PV in later stages does not mean oxidative rancidity has stopped; rather, it indicates the transition from primary oxidation products to secondary ones (e.g., aldehydes, ketones), which are responsible for the characteristic off-flavours and odours.

### Enzymatic activity profiles during storage-induced rancidity in pearl millet flour

**Figure 5:**
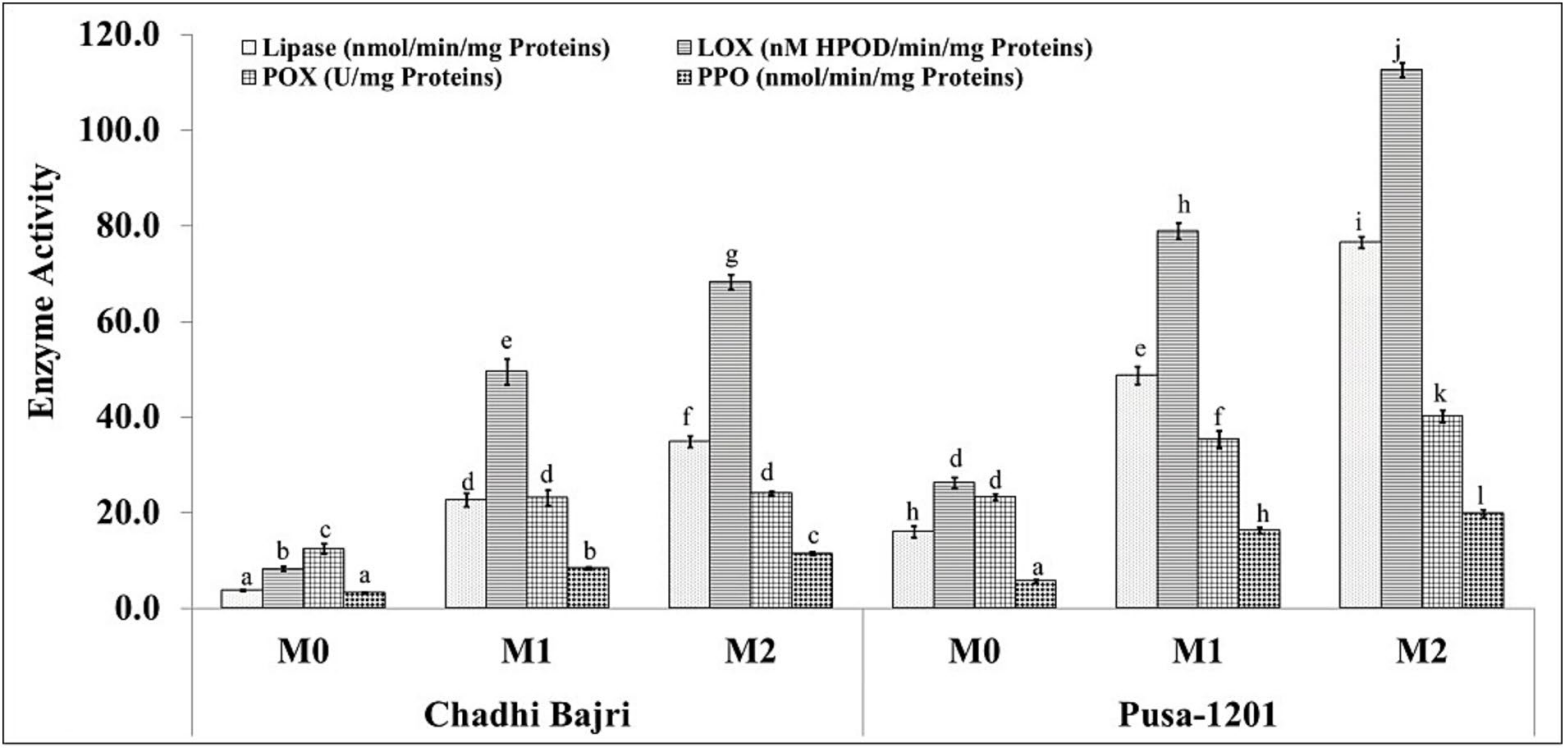
Changes in the activities of rancidity causing enzymes in Chadhi Bajri and Pusa-1201 flours during storage. **(a)** Lipase activity, **(b)** lipoxygenase (LOX) activity, **(c)** peroxidase (POX) activity, and (d) polyphenol oxidase (PPO) activity. Data are presented for fresh (M_0_), 1-month (M_1_), and 2-month (M_2_) stored samples. Values are expressed as mean ± standard deviation (*n* = 3). Statistical significance between groups at different time points is indicated by different letters above histogram.

The activities of key enzymes associated with lipid degradation and oxidative deterioration—lipase, lipoxygenase (LOX), peroxidase (POX), and polyphenol oxidase (PPO)—were quantified in fresh (M_0_), 1-month (M_1_), and 2-month (M_2_) stored flour samples of two pearl millet cultivars, Chadhi Bajri and Pusa-1201 (Figure 5). In Chadhi Bajri, lipase activity increased significantly from ~5 nmol/min/mg protein in M_0_ to ~25 nmol/min/mg in M_1_ and ~40 nmol/min/mg in M_2_, indicating progressive triglyceride hydrolysis with storage. A similar trend was observed for LOX activity, which rose from ~15 to ~50 and ~70 nM HPOD/min/mg protein in M_0_, M_1_, and M_2_, respectively_._ POX activity also increased from ~10 U/mg protein in M_0_ to ~20 U/mg in M_1_ and M_2_, while PPO activity remained comparatively lower, peaking at ~15 nmol/min/mg protein in M_1_ before declining in M_2_.

In contrast, Pusa-1201 exhibited markedly higher enzyme activities across all storage stages. Lipase activity surged from ~15 nmol/min/mg in M_0_ to ~50 and ~90 nmol/min/mg in M_1_ and M_2_, respectively. LOX activity displayed a steep increase, reaching ~75 and ~110 nM HPOD/min/mg protein in M_1_ and M_2_, respectively, the highest among all measured enzymes. POX activity increased steadily from ~15 to ~35 U/mg protein, while PPO activity rose to ~25 nmol/min/mg protein by M_2_. Sinha et al. (55) reported the roles of lipase and LOX in rancidity and suggested several strategies to reduce enzymatic deterioration in rice bran.

These results highlight a clear cultivar-dependent difference in enzymatic response to storage, with Pusa-1201 showing more aggressive lipid degradation and oxidative enzyme activity, correlating well with its higher rancidity markers. The elevated levels of lipase and LOX, in particular, suggest their pivotal role in accelerating rancidity, while the concurrent rise in POX and PPO may contribute to browning and further oxidative deterioration. Li et al. (56) demonstrated that processing of grains reduces the lipase and peroxidase activities, and is correlated with the aroma evolution during storage. Kumar et al. (7) provides direct insight into lipase, LOX, POX, and PPO dynamics in pearl millet, emphasising their roles in rancidity and storage quality.

### Correlation of lipid oxidation-associated metabolites with AV and PV in flour

A clear association was observed between the accumulation of rancidity-linked metabolites and the measured acid value (AV) and peroxide value (PV) in stored pearl millet flours. In both Chadhi Bajri and Pusa-1201, progressive storage from fresh (M_0_) to 1-month (M_1_) and 2-month (M_2_) conditions resulted in significant increases in AV and PV, reflecting elevated levels of hydrolytic and oxidative lipid degradation, respectively. This biochemical deterioration correlated with enhanced activities of lipase, lipoxygenase (LOX), and other oxidative enzymes, as well as the accumulation of specific lipid-derived metabolites detected through metabolomic profiling. Vinutha et al. (57) demonstrated that lipase and LOX inactivation significantly reduced FFA accumulation and PV during storage, underscoring the causal link between enzyme activity and AV/PV escalation.

Metabolites such as linoleic acid, oleic acid, palmitic acid, 2-octenal, hexanal, and nonanal—known products of enzymatic and non-enzymatic oxidation—showed strong positive correlation with increasing PV values, indicating active lipid peroxidation processes mediated by LOX. These volatile aldehydes and hydroperoxides are typical secondary oxidation products contributing to off-flavour and odour in rancid flour. The increase in free fatty acids (FFAs) such as oleic and linoleic acids also aligned with rising AV values, corroborating the lipase-driven hydrolysis of triglycerides during storage. Yogendra et al. (29) observed significant correlations between FFAs/unsaturated FAs and acid value (AV), as well as between lipid hydroperoxides and peroxide value (PV).

In Pusa-1201, which displayed a higher degree of rancidity, both AV and PV were significantly elevated compared to Chadhi Bajri, matching the elevated abundance of oxidised lipid derivatives and FFA metabolites. Additionally, the presence of oxidised sterol fragments, epoxy fatty acids, and ketones further supported the advanced stage of lipid degradation in M_2_ samples. These findings suggest that metabolite profiling not only mirrors but also mechanistically supports the enzymatic and chemical processes measured through AV and PV. Bai et al. (58) showed that storage led to marked increases in AV, PV, free fatty acids, and volatile aldehydes such as hexanal, aligning with LOX activity and off-odour development. Overall, the metabolomic signatures strongly corroborate the biochemical indices of rancidity, establishing a direct biochemical link between enzymatic activity, lipid and rancidity.

### Correlation of metabolite signatures with rancidity causing enzymes during flour storage

The metabolomic profiling of pearl millet flour revealed distinct accumulation patterns of lipid-derived and oxidative metabolites that correlated strongly with the enzymatic activity of lipase, lipoxygenase (LOX), peroxidase (POX), and polyphenol oxidase (PPO) during storage. The increase in free fatty acids (FFAs) such as oleic acid, linoleic acid, and palmitic acid was closely aligned with elevated lipase activity, especially in M_1_ and M_2_ samples of both Chadhi Bajri and Pusa-1201. This indicates that lipase-mediated hydrolysis of triacylglycerols is a primary driver of rancidity with the released FFAs serving as precursors for further oxidative degradation. Our findings are in conformity with the observation of (59), who established the correlation between the lipase activity to FFA accumulation and rancidity in pearl millet.

Simultaneously, high LOX activity corresponded with increased levels of lipid hydroperoxides and downstream aldehydic volatiles such as hexanal, nonanal, and 2-octenal, which are typical markers of linoleic acid peroxidation (7). These compounds were particularly abundant in the more rancid Pusa-1201 cultivar, reflecting the cultivar’s heightened LOX activity and oxidative susceptibility. The presence of epoxy fatty acids, hydroxy- and keto-derivatives, and short-chain aliphatic aldehydes also pointed towards enzymatic oxidation routes facilitated by LOX (60).

Moreover, elevated POX and PPO activities showed strong association with the accumulation of phenolic-derived quinones, oxidised flavonoid fragments, and brown-coloured polymeric products, especially in M_2_ samples. These oxidative enzymes likely contributed to non-lipid oxidation and browning reactions that accompany rancidity, particularly under prolonged storage. Their activities were more pronounced in Pusa-1201, mirroring the higher abundance of oxidation-derived metabolites and suggesting a synergistic role of enzymatic browning and lipid oxidation in driving flour deterioration. The finding is in conformity with the observation of (61), who reported that PPO activity oxidises phenolics to o-quinones, which non-enzymatically polymerise into brown pigments and highlight the dual role of POX in further oxidising phenolic substrates using hydrogen peroxide, intensifying browning.

Collectively, the metabolite patterns showed the functionality of several enzymes, establishing the fact that rancidity development is a multi-enzyme mediated process involving lipid hydrolysis (lipase), fatty acid peroxidation (LOX), and phenolic oxidation (POX, PPO). These metabolite-enzyme correlations provide molecular insights into cultivar-specific rancidity mechanisms and can help in devising strategies for rancidity mitigation through genetic or post-harvest interventions.

Various literatures have proposed non-thermal and thermal methods to enhance the shelf-life of pearl millet flour. Non-thermal methods like addition of salt, lime treatment, soaking and germination has been reported to enhance the shelf life up to 3 months (5). Similarly, thermal methods like heating, microwave treatment, steaming, hydrothermal and HT-IR have bene reported to be effective in enhancing the shelf-life of the flour by 3 months, though with compromised quality (7, 62). Therefore, there is a need to develop rapid, cost-effective technologies that can enhance the keeping quality of pearl millet flour without altering its native food matrix.

The present study advances rancidity research in pearl millet flour by integrating untargeted LC–MS metabolomics with enzymatic profiling, thereby moving beyond conventional biochemical indices such as acid value, peroxide value, and free fatty acid content. This combined approach enabled the discovery of 25 discriminatory metabolites, including phytol, ethanolamine, chlorophyllide b, nucleotide derivatives, and glucoside derivatives, which serve as robust biomarkers for rancidity. A key novelty lies in linking rancidity progression with nucleic acid degradation and disrupted energy metabolism, as evidenced by the accumulation of guanine, UTP, xanthosine, and deoxycytidine. Comparative metabolomic analysis of a high-yielding hybrid (Pusa-1201) and a landrace (Chadhi Bajri) further revealed cultivar-specific biochemical signatures, where the landrace accumulated antioxidant and stress-protective metabolites (quercetin 3-sulfate, rosmarinate), conferring greater resistance to rancidity. The strong correlations established between enzyme activities (lipase, LOX, POX, PPO) and rancidity-linked metabolites highlight rancidity as a multi-enzyme mediated process, offering mechanistic clarity lacking in earlier studies. Collectively, these findings provide a superior framework compared to previous reports by identifying metabolite-enzyme signatures of rancidity, elucidating cultivar-dependent susceptibility, and offering potential biomarkers for the development of rapid rancidity sensors in pearl millet.

Identified biomarkers associated with rancidity, such as specific lipase gene variants, transcripts, or enzyme activity levels, can serve as powerful screening tools in pearl millet breeding programs (59). By correlating these biomarkers with low rancidity phenotypes, breeders can rapidly and accurately identify lines that are less prone to lipid degradation without waiting for long-term storage tests. Molecular markers linked to low-lipase activity or reduced free fatty acid accumulation can be used in marker-assisted selection (MAS), enabling the early selection of desirable genotypes at the seedling stage. Similarly, biochemical markers, such as metabolite signatures indicative of oxidative stability, can be measured in developing grains or flour to predict shelf-life potential. Using these biomarkers accelerates breeding for improved storage quality, reduces reliance on time-consuming post-harvest assays, and allows simultaneous selection for other agronomic traits, ultimately facilitating the development of pearl millet *cvs*. with superior flour stability and consumer-acceptable quality.

## Conclusion

This study elucidates the metabolic and enzymatic basis of rancidity development in pearl millet flour, focusing on two cultivars – Pusa-1201 and Chadhi Bajri. Using a metabolomics approach, significant alterations in metabolite profiles were observed during storage, revealing cultivar-dependent susceptibility to rancidification. In Pusa-1201, a sharp increase in key metabolites such as D-Glucono-1,5-lactone 6-phosphate (177.9-fold), triphosphate (80.2-fold), and UTP (63.9-fold) indicated disruption in carbohydrate and energy metabolism. Accumulation of ethanolamine, phytol, and fatty acid derivatives like trans-Oct-2-enoyl-[ACP] signalled enhanced lipid oxidation. Pigment degradation was marked by increased chlorophyllide b and pyropheophorbide a, reflecting oxidative damage. Comparative analysis between Pusa-1201 Rancid and Chadhi Bajri Rancid flours further confirmed varietal differences, with Pusa-1201 showing elevated levels of nucleotide degradation products (xanthosine, deoxycytidine) and lipid oxidation markers such as 3-oxotetradecanoyl-CoA and C25-allenic-apo-aldehyde. Enzyme activity profiling revealed a time-dependent increase in lipase, lipoxygenase (LOX), peroxidase (POX), and polyphenol oxidase (PPO), which positively correlated with rancidity indices—acid value (AV) and peroxide value (PV)—and metabolite accumulation. Lipase was linked to FFA release, initiating hydrolytic rancidity, while LOX activity promoted peroxidation and volatile aldehyde formation. POX and PPO contributed to oxidative browning and degradation. Together, these data demonstrate that rancidity in pearl millet flour is driven by coordinated enzymatic activity and metabolic breakdown, with Pusa-1201 being more prone to deterioration than Chadhi Bajri. Since, the present findings has been generated using only two *cvs*., there is a need to characterise large number of diverse *cvs*. in order to develop a datasets. The integration of metabolomic and enzymatic analyses offers potential biomarkers for early detection and provides a foundation for selecting low-rancidity genotypes.

## Data Availability

The original contributions presented in the study are included in the article/Supplementary material, further inquiries can be directed to the corresponding author.
