# Supplementary figures and images for: Characterising the enzyme-driven metabolic shifts in rancid pearl millet flour using metabolomics approaches: a step towards improving quality and shelf-life
Source: Front Nutr. 2025 Oct 23;12:1691522. doi: 10.3389/fnut.2025.1691522 (PMC12588842; doi:10.3389/fnut.2025.1691522)

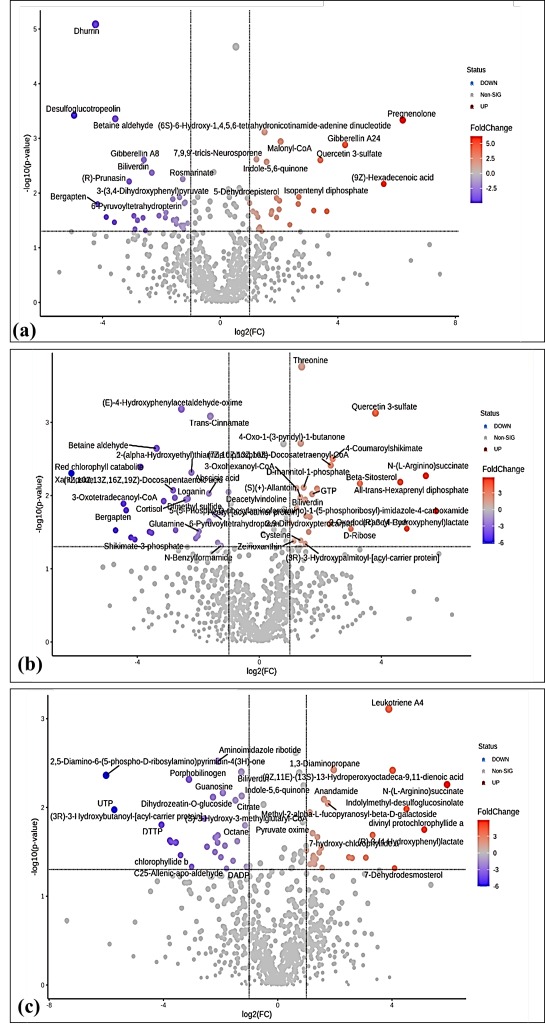

Supplement: Supplementary file 7 [file Image_1.JPEG]

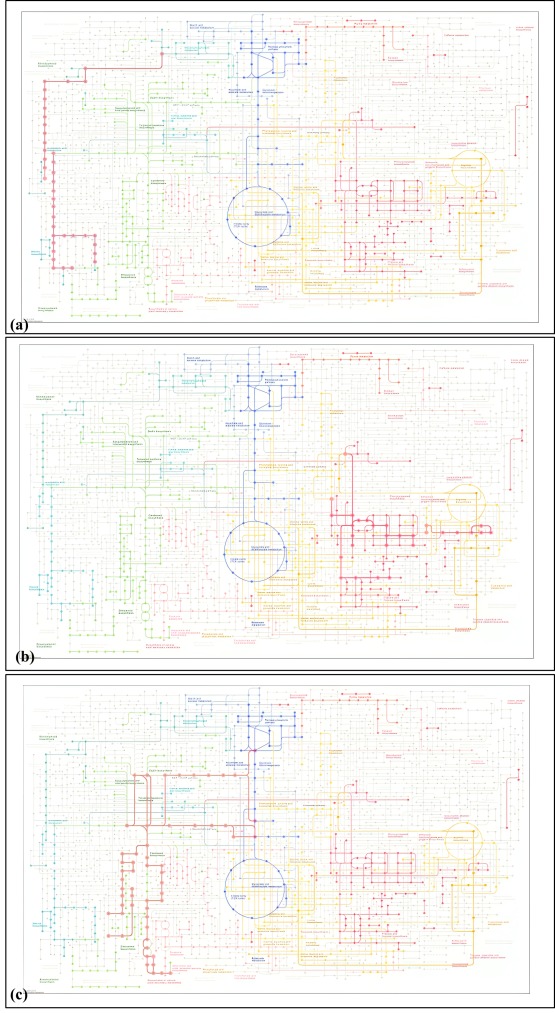

Supplement: Supplementary file 8 [file Image_2.JPEG]
